# The costs of negative affect attributable to alcohol consumption in later life: A within-between random longitudinal econometric model using UK Biobank

**DOI:** 10.1371/journal.pone.0211357

**Published:** 2019-02-13

**Authors:** Chenlu Li, Simon C. Moore, Jesse Smith, Sarah Bauermeister, John Gallacher

**Affiliations:** 1 Department of Psychiatry, Warneford Hospital, University of Oxford, Oxford, United Kingdom; 2 Crime and Security Research Institute, Friary House, Greyfriars Road, Cardiff, United Kingdom; 3 Violence Research Group, School of Dentistry, Cardiff University, Cardiff, United Kingdom; 4 Centre for the Development and Evaluation of Complex Interventions for Public Health Improvement, School of Social Sciences, Cardiff University, Cardiff, United Kingdom; University of Sao Paulo Medical School, BRAZIL

## Abstract

**Aims:**

Research demonstrates a negative relationship between alcohol use and affect, but the value of deprecation is unknown and thus cannot be included in estimates of the cost of alcohol to society. This paper aims to examine this relationship and develop econometric techniques to value the loss in affect attributable to alcohol consumption.

**Methods:**

Cross-sectional (n = 129,437) and longitudinal (n = 11,352) analyses of alcohol consumers in UK Biobank data were undertaken, with depression and neuroticism as proxies of negative affect. The cross-sectional relationship between household income, negative affect and alcohol consumption were analysed using regression models, controlling for confounding variables, and using within-between random models that are robust to unobserved heterogeneity. The differential in household income required to offset alcohol’s detriment to affect was derived.

**Results:**

A consistent relationship between depression and alcohol consumption (β = 0.001, z = 7.64) and neuroticism and alcohol consumption (β = 0.001, z = 9.24) was observed in cross-sectional analyses, replicated in within-between models (depression β = 0.001, z = 2.32; neuroticism β = 0.001, z = 2.33). Significant associations were found between household income and depression (cross sectional β = -0.157, z = -23.86, within-between β = -0.146, z = -9.51) and household income and neuroticism (cross sectional β = -0.166, z = -32.02, within-between β = -0.158, z = -7.44). The value of reducing alcohol consumption by one gram/day was pooled and estimated to be £209.06 (95% CI £171.84 to £246.27).

**Conclusions:**

There was a robust relationship between alcohol consumption and negative affect. Econometric methods can value the intangible effects of alcohol use and may, therefore, facilitate the fiscal determination of benefit.

## Introduction

The potential benefits of light alcohol consumption has long been described in terms of greater happiness, reduced anxiety and positive changes in other affective states [1]. The pursuit of happiness is an unalienable right in some jurisdictions [2] and the UK Government explicitly referenced the presumed positive effect of alcohol on well-being in the 2012 Alcohol Strategy [3]. However, there is a reliable and consistent relationship between greater alcohol use and negative affect [3]. Initiatives that reduce alcohol consumption should, therefore, be expected to elicit improvements to a population’s affective state.

Although the directionality of the relationship between alcohol use and affect has historically been difficult to determine, attention to possible underlying mechanisms suggests that greater alcohol use results in a net detriment to the consumer’s affective state, or personal utility. Unidirectional models typically fit data more accurately than bidirectional models where personal utility also influences alcohol use [4, 5]. Models also suggest a J-shaped association curve between consumption and various measures of personal utility. For example, depression is lowest, and happiness is greatest, in light drinkers [6–10]; however, moderate and heavy consumption of alcohol, even in the absence of a disorder, tends to reverse any protective effect and increase the risk of mental health problems to a level above that observed in those who abstain [6–8]. In short, abstaining appears to be a risk factor for decreased personal utility, but moderate or heavy alcohol consumption is a higher risk factor. Some explain this trend as the “sick quitter” effect, which occurs when a subset of abstainers refrains from drinking due to other health factors that increase their risk of adverse effects. These factors serve as latent confounders and falsely inflate the risk attributable to abstaining [11]. Further, in the population suffering from comorbid depression and alcohol use disorder or dependence, alcohol problems are typically the initial disorder [4, 5].

One mechanism underlying this relationship may include a role for alcohol in circadian dysfunction. Even in moderate amounts, alcohol has been shown to disrupt the circadian rhythm and interrupt transcription of regulatory genes in related networks, including CLOCK, *CRY1*, *CRY2*, *ARNTL*, & *PER2* [12–15]. Circadian-related single nucleotide polymorphisms have been associated with comorbid depression and alcohol abuse [16]. Alcohol consumption has also been observed to lower serotonin levels and morph the shape of the concentration curve to mimic that found in those diagnosed with depression [14]. This body of evidence points to alcohol as a causal factor in circadian dysregulation, which in turn may progress depression via potentiation of risk factors for depression including serotonin levels, altered emotional regulation, or deficits in sociality. Another possible mechanism centres on brain-derived neurotrophic hormone (*BNDF*), a stimulator of neurogenesis and necessary for the growth and repair of neural cells. Altered *BDNF* expression has been a prime candidate in the epigenetic exploration of depression. In adolescent rats, binge drinking is associated with a reduction in *BDNF* expression, which is in turn associated with decreased survival time of neural progenitor cells and the display of a depressive phenotype [17]. In sum, the weight of observational, genetic and neuroscientific evidence together indicates that increasing levels of alcohol consumption causes a deterioration in personal utility.

Studies are increasingly seeking to capture the intangible benefits of initiatives and interventions that seek to improve health [18–20], anxiety and depression [21], and are doing so in monetary terms [22–25]. For alcohol, however, the value of reducing consumption has typically focussed on quantifiable outcomes such as the price of hospital admission and costs to economic productivity [26–28]; intangible costs and benefits are less prominent in estimates. This focus is notable, as the presumed affective benefits of alcohol have been used to counterbalance policies aimed at reducing alcohol consumption and therefore harm [3]. Policymakers and consumers may, therefore, ask whether the cost of any reduction in alcohol consumption on affect is worthwhile. Policymakers, for example, may consider interventions to improve personal utility in populations through targeting interventions at alcohol consumption and may wish to know whether efforts are cost-effective. Consumers, meanwhile, may lack the necessary information to compare the consequences of alcohol, such as negative affect, with consequences of other consumption decisions. Money is, by nature, fungible and exchangeable for a range of goods and services. Providing a value for the affective consequences of alcohol use allows consumers the option to consider the combined financial, health and personal utility costs of their alcohol use and consider ways to invest their resources to maximise personal utility. Placing a value on the personal utility consequences of alcohol use facilitates this decision-making for both policy makers and consumers.

Shadow pricing methods [29] can be used to determine the price of non-market goods [30], including the affective benefits derived from clean air and green landscapes [31], the value of fear in response to crime [32] and aircraft noise [33]. The method is derived from observations that wages often vary systematically by the unpleasantness of employment, that is, that a higher wage is necessary to offset occupations that are unpleasant to compensate and therefore attract employees [34, 35]. With respect to alcohol consumption, personal utility (*U*) can be expressed as a function of affective state. Alcohol consumption is associated with a reduction in *U* but that *U* increases as household income increases [36–38]. We therefore define the shadow price of alcohol as the compensating differential, the increase in household income, required to offset the effect of an alcohol-induced detriment in affect.

In sum, the aims of this work are, first, to determine the nature and relationship between alcohol consumption and personal utility, and, second, to apply econometric models to estimate the relative value of any change. This investigation uses data from UK Biobank, a prospective cohort study based in the United Kingdom (UK) [39]. Biobank records a wealth of data from respondents, aged 37 to 73 years of age, including alcohol use, and includes two measures relating to affect. A neuroticism scale [40] describing levels of anxiety and depression, and a brief depression scale [41]. Depression is associated with panic, social anxiety, and post-traumatic stress disorder [42] whereas neuroticism [40] is an aspect of personality that is not only associated with anxiety and depression, but further predicts mortality in older men [43]. Further, a smaller group of respondents were followed up using the measures of interest so providing an opportunity to consider both between and within changes. For completeness, we use both scales each in cross-sectional and longitudinal analysis, excepting that the reduced numbers in the longitudinal analyses may leave it underpowered. We undertake analyses in parallel. We consider results from both longitudinal and cross-sectional analyses for both depression and neuroticism in deriving estimates. We restrict analyses to only those consuming alcohol as the reasons for abstinence cannot be accounted for in models.

## Methods

### Study participants

UK Biobank participants (N = 502,647) are aged between 37 and 73 years and were recruited at 22 assessment centres across the United Kingdom (UK) between 2006 and 2010 [39]. Participants undertook comprehensive mental health, cognitive, lifestyle, biomedical, demographic and physical assessment and measurement. Only participants who reported that they consumed alcohol were included in the current study, as the study requires alcohol consumers by definition. This eligibility requirement also addressed possible “sick quitter” effects in equations. All participants gave written informed consent, and participants who later withdrew from Biobank (n = 31) were excluded from analyses. Ethical approval was granted to Biobank from the NHS Health Research Authority, Research Ethics Committee (North West, Haydock Research Ethics Committee, Manchester, M1 3DZ, UK; reference 11/NW/0382). The current study was conducted using the UK Biobank Resource (application number 15008). There are restrictions prohibiting the provision of data in this paper. The data were obtained from a third party, UK Biobank, upon application. Interested parties can apply for data from UK Biobank directly (www.biobank.ac.uk). UK Biobank will consider data applications from bona fide researchers for health-related research that is in the public interest. By accessing data from UK Biobank, readers will be obtaining it in the same manner as the authors. The authors had no special access privileges to the data.

### Materials

Participants completed a selection of mental health scales presented on a touchscreen computer, including the Patient Health Questionnaire four-item version (PHQ-4) [41] to capture depression. The PHQ-4 encompasses core criteria for depressive disorders [42], which have also been shown to be useful screening items for panic, social anxiety, and post-traumatic stress disorders [42]. Participants were required to rate, on a four-point Likert scale from zero (not at all) to three (nearly every day), their response to four items: “frequency of depressed mood”, “frequency of unenthusiasm / disinterest”, “frequency of tenseness / restlessness” and “frequency of tiredness / lethargy”. An item response theory (IRT) graded response model was applied to the 4-item graded PHQ-4 scale, a depression latent theta metric *Θ*_*D*_ was derived for each participant and used as the outcome in the analyses reported here. A positive *Θ*_*D*_ suggests possession or endorsement of depression and a negative *Θ*_*D*_ suggests absence or non-endorsement with most respondents recording values between -3 and +3 [44]. In comparison with a summated score, *Θ*_*D*_ is less likely to over-estimate repeated measure variance [45], produces smaller sample bias [46] and provides additional individual scale item-level information.

The 12-item neuroticism EPQ-R scale [47, 48] measures the neurotic dimension of personality, which indicates emotional instability and reactiveness. Participants were required to answer, “yes”, “no”, “I don’t know” or “I do not wish to answer” in response to the 12 questions: “Does your mood often go up and down?”, “Do you ever feel just miserable for no reason?”, “Are you an irritable person?”, “Are your feelings easily hurt?”, “Do you often feel fed-up?”, “Would you call yourself a nervous person?”, “Are you a worrier?”, “Would you call yourself tense or highly strung?”, “Do you worry too long after an embarrassing experience?”, “Do you suffer from nerves?”, “Do you often feel lonely?”, and “Are you often troubled by feelings of guilt?” A two-parameter IRT model was applied to the 12-item binary response item scale using methods already described for depression and a post-estimation neuroticism latent theta metric (*Θ*_*N*_) was derived for each participant.

Alcohol consumption data were derived from the questions about alcohol intake frequency and beverage type. Drinking frequency categories are based on the following question: “about how often do you drink alcohol?”, and possible answers are: “daily or almost daily”, “three or four times a week”, “once or twice a week”, “one to three times a month”, “special occasions only”, “never”, and “prefer not to answer.” Among alcohol drinkers, questions were asked about the amount consumed weekly or monthly for the following beverage types: pints of beer or cider including bitter, lager, stout, ale and Guinness; glasses of white wine or champagne; glasses of red wine; glasses of fortified wine including sherry, port and vermouth; standard measure (35ml) of spirits or liqueurs including whisky, gin, rum, vodka and brandy; and glasses of other alcoholic drinks. For weekly (monthly) drinkers, the total grams of alcohol consumed per week (month) was calculated as the summation of the multiplication of the average number of alcoholic drinks consumed each week (month) by the average grams of alcohol contained in each type of drink using the UK Food Standard Agency’s guidelines [49]. Weekly (monthly) alcohol intake is divided by seven (30.44 for monthly drinkers) to obtain averaged daily alcohol intake.

Categorical information on annual household income is available in Biobank: less than £18,000; £18,000 to £30,999; £31,000 to £51,999; £52,000 to £100,000 and greater than £100,000. Due to the unequal distribution of these bands, the natural logarithm of household income is used as a cardinal approximation to household income.

Educational attainment was included as a binary variable (with or without a university or college degree) as was tobacco use status (smoker or non-smoker), ethnicity (white or other), long-term illness (with or without), and consumption of fruit and vegetables (below 5 portions a day compared to equal or above). Socioeconomic deprivation was assessed using the continuous Townsend deprivation score where a higher score implies greater material deprivation [50]. Age was included as a continuous variable. Body mass index (BMI) was included as two binary variables with normal weight defined as ≤ 24.9kg/m^2^ compared to overweight 25.0 to 29.9kg/m^2^ and obese ≥ 30kg/m^2^.

### Analytic sample

In the cross-sectional analysis, participants who chose to respond “I don’t know” or “I do not wish to answer” to any of the four items of the depression scale or the 12 items of the neuroticism scale at baseline were excluded from analyses (n = 129,437). Participants who self-reported a neurological condition were excluded from analyses (n = 8,213) [51], as were non-drinkers (n = 68,150). Participants with a self-reported history of alcohol-related problems or dependency (n = 407) were also dropped due to some evidence for a bidirectional effect in clinically relevant groups [52] and the risk of unmeasured externalities affecting estimates. Case-wise deletion was applied to all demographic variables (n = 53,683 were excluded). The total number of participants available for these analyses were 243,133 (n = 125,792 male, aged 40–74 years).

In the longitudinal analysis, participants who chose to respond, “I don’t know” or “I do not wish to answer” to any of the four items of the depression scale or the 12 items of the neuroticism scale at both baseline and first assessment were excluded from analyses. Participants who self-reported a neurological condition were excluded from analyses (n = 461) [51], as were participants with a self-reported history of alcohol-related problems/dependency (n = 19). Case-wise deletion was applied to all demographic variables at both baseline and first assessment (n = 1,992 excluded). Balanced panel data was available for a sample of 11,352 respondents (n = 5,885 male, aged between 40 and 74 years). Descriptive statistics are reported in Table 1.

**Table 1:** Descriptive statistics for the depression and neuroticism equations.

	Baseline N = 243,133				First assessment N = 11,352				Longitudinal N = 11,352	
	%	${\overset{-}{\Theta }}_{D}$ (SD)	${\overset{-}{\Theta }}_{N}$ (SD)	$\overset{-}{AC}$ (SD)	%	${\overset{-}{\Theta }}_{D}$ (SD)	${\overset{-}{\Theta }}_{N}$ (SD)	$\overset{-}{AC}$ (SD)	*f*(*Θ*_*D*_) (*z*)	*f*(*Θ*_*N*_) (*z*)
**Household Income**										
<£18,000	0.167	0.052 (0.878)	0.098 (0.918)	20.987 (22.828)	0.158	0.060 (0.852)	0.098 (0.929)	12.878 (17.419)	Reference	
(£18,000, £30,999]	0.240	-0.083 (0.782)	-0.025 (0.883)	20.355 (19.653)	0.301	-0.077 (0.750)	-0.006 (0.881)	14.085 (16.193)	-0.10*** (-4.87)	-0.09*** (-3.92)
(£31,000, £51,999]	0.277	-0.078 (0.758)	-0.044 (0.873)	21.062 (19.354)	0.286	-0.055 (0.740)	-0.018 (0.868)	16.053 (17.152)	-0.08*** (-4.10)	-0.11*** (-4.72)
(£52,000, £100,000]	0.244	-0.102 (0.724)	-0.102 (0.860)	21.491 (18.321)	0.204	-0.049 (0.735)	-0.042 (0.873)	17.033 (17.084)	-0.08*** (-3.71)	-0.14*** (-5.54)
>£100,000	0.072	-0.178 (0.679)	-0.226 (0.838)	22.569 (17.790)	0.050	-0.150 (0.659)	-0.181 (0.830)	17.474 (14.690)	-0.17*** (-6.27)	-0.27*** (-7.77)
**Education**										
Other	0.610	-0.056 (0.800)	-0.004 (0.887)	21.798 (20.928)	0.509	-0.041 (0.776)	0.018 (0.901)	15.461 (17.685)	Reference	
College	0.390	-0.095 (0.738)	-0.104 (0.869)	19.990 (17.576)	0.491	-0.053 (0.740)	-0.037 (0.862)	14.987 (15.935)	-0.02 (-1.84)	-0.06*** (-4.20)
**Gender**										
Female	0.483	-0.011 (0.784)	0.098 (0.860)	14.202 (12.430)	0.482	0.016 (0.777)	0.116 (0.877)	9.522 (10.788)	Reference	
Male	0.517	-0.127 (0.761)	-0.175 (0.881)	27.521 (22.826)	0.518	-0.105 (0.736)	-0.125 (0.873)	20.529 (19.524)	-0.12*** (-9.93)	-0.25*** (-15.72)
**Employment**										
Other	0.363	-0.136 (0.782)	-0.059 (0.889)	20.809 (20.128)	0.552	-0.108 (0.744)	-0.049 (0.876)	15.152 (16.759)	Reference	
Employed	0.637	-0.034 (0.767)	-0.034 (0.877)	21.254 (19.464)	0.448	0.028 (0.769)	0.039 (0.889)	15.322 (16.961)	0.14*** (12.17)	0.08*** (5.40)
**Smoking Status**										
No	0.901	-0.095 (0.757)	-0.056 (0.875)	20.034 (18.388)	0.958	-0.054 (0.751)	-0.012 (0.880)	14.895 (16.361)	Reference	
Yes	0.099	0.147 (0.884)	0.079 (0.925)	30.775 (27.288)	0.042	0.127 (0.891)	0.062 (0.945)	22.783 (24.352)	0.18*** (5.53)	0.07 (1.85)
**Vegetable and fruit**										
< 5 unit per day	0.282	0.008 (0.813)	0.013 (0.900)	24.529 (23.116)	0.289	0.015 (0.800)	0.029 (0.908)	17.659 (19.616)	Reference	
≥ 5 unit per day	0.718	-0.102 (0.756)	-0.065 (0.873)	19.745 (18.020)	0.711	-0.072 (0.739)	-0.025 (0.872)	14.238 (15.474)	-0.09*** (-6.76)	-0.06*** (-4.02)
**Ethnicity**										
Other	0.030	0.110 (0.890)	-0.049 (0.904)	14.499 (17.167)	0.017	0.108 (0.964)	-0.060 (0.922)	7.060 (12.412)	Reference	
White	0.970	-0.076 (0.770)	-0.043 (0.881)	21.296 (19.750)	0.983	-0.049 (0.754)	-0.008 (0.882)	15.371 (16.881)	-0.16** (-2.63)	0.06 (0.91)
**BMI**										
Normal weight	0.343	-0.092 (0.757)	0.001 (0.876)	18.259 (17.164)	0.373	-0.077 (0.741)	0.010 (0.877)	13.078 (14.145)	Reference	
Overweight	0.442	-0.101 (0.762)	-0.084 (0.878)	22.323 (19.957)	0.426	-0.080 (0.740)	-0.043 (0.880)	16.744 (17.497)	-0.00 (-0.31)	-0.07*** (-4.47)
Obese	0.216	0.024 (0.817)	-0.030 (0.892)	23.074 (22.304)	0.201	0.081 (0.815)	0.027 (0.900)	16.001 (19.454)	0.16*** (8.97)	0.01 (0.35)
**Long-term illness**										
No	0.724	-0.138 (0.736)	-0.098 (0.862)	20.848 (18.971)	0.701	-0.121 (0.713)	-0.064 (0.862)	15.664 (16.803)	Reference	
Yes	0.276	0.106 (0.842)	0.101 (0.914)	21.736 (21.508)	0.299	0.129 (0.830)	0.121 (0.916)	14.206 (16.916)	0.24*** (17.73)	0.18*** (11.11)
	*Mean* *(SD)*				*Mean* *(SD)*					
**Age**	56.494 (8.000)				57.086 (7.415)				-0.001*** (-20.47)	-0.001*** (-15.21)
**Townsend**	-1.642 (2.861)				-2.123 (2.603)				0.02*** (9.30)	0.02*** (5.81)
**No. of children**	1.770 (1.180)				1.739 (1.184)				-0.04*** (-6.84)	-0.05*** (-7.01)

### Analytic strategy

Formally, utility, *U*, is described as a linear function of household income *Y* and alcohol consumption *AC*,

U=f(lnY,AC) (1)

If it holds that as *Y* increases *U* decreases, then it is possible to calculate the additional income necessary to offset the consequences of variables that decreases *U*. The differential of *U* is:

$$\Delta U=\frac{\partial U}{\partial lnY}\Delta lnY+\frac{\partial U}{\partial AC}\Delta AC$$ (2)

On the indifference curve, *ΔU = 0*, therefore, variation in log household income is

$$\Delta lnY=-\frac{\partial U/\partial AC}{\partial U/\partial lnY}\Delta AC$$ (3)

The compensating variation in household income (*CIV*) is:

$$\Delta Y=Y*(\text{exp}\left(-\frac{\partial U/\partial AC}{\partial U/\partial lnY}\right)*\Delta AC-1)$$ (4)

A within-between random model is applied to data from baseline to the first assessment. Fixed- and random-effects models are typically applied in hierarchically clustered data analysis. Random-effects models refer to models for clustered data that have both random effects and fixed effects, also known as multilevel models or mixed models. Fixed effects models refer to those for clustered data that allow for arbitrary dependence between the unobserved effects and the covariates [53]. On the one hand, a fixed-effect model provides consistent estimates of within-cluster variation effects (level one), even if there is unobserved heterogeneity at the cluster level. It cannot estimate the effect of any variable that does not vary within clusters and that holds for all level two variables. On the other hand, a random effect allows for estimation of the effect of cluster-invariant variables (level two) on the outcome. However, it relies on the assumption of strictly exogenous regressors with respect to the idiosyncratic error component. The within-between random model, which combines the strengths of the two, has gained attention recently [54–60].

In the longitudinal analysis, repeated measures at level one, which is denoted by subscript *t*, are clustered in individuals at level two, denoted by subscript *i*. Specifically, the multi-variate within-between random model applied in this analysis is given as follows:

$$\Theta_{i,t}=\alpha +\sum_{k=1}^{K}\beta_{k}^{w}(x_{i,k,t}-{\bar{x}}_{i,k})+\sum_{k=1}^{K}\beta_{k}^{b}{\bar{x}}_{i,k}+\sum_{j=1}^{J}\beta_{j}c_{i,j}+v_{i}+\epsilon_{i,t}$$ (5)

Where *x*_*i*,*k*,*t*_ is the *k*^th^ level-one variable that varies between and within cluster; ${\bar{x}}_{i,k}$ is the cluster mean,

$$n_{i,k}^{-1}\sum_{t=1}^{n_{i,k}}x_{i,k,t}$$ (6)

the between component; *c*_*i*,*j*_ is the *j*^th^ level-two variable that varies only between clusters; *v*_*i*_ is the level-two error and random intercept in the within-between random model; and *ϵ*_*i*,*t*_ is the level-one error.

Eq 5 can be rearranged into a correlated random-effect model [61]:

$$\Theta_{i,t}=\alpha +\sum_{k=1}^{K}\beta_{k}^{w}x_{i,k,t}+\sum_{j=1}^{J}\beta_{j}^{R}c_{i,j}+\left(\sum_{k=1}^{K}\left(\beta_{k}^{b}-\beta_{k}^{w}\right){\bar{x}}_{i,k}+v_{i}\right)+\epsilon_{i,t}$$ (7)

$\mu_{i}=\sum_{k=1}^{K}\left(\beta_{k}^{b}-\beta_{k}^{w}\right){\bar{x}}_{i,k}+v_{i}$ introduces the assumption that *μ*_*i*_, the level-two error in a random-effect model, depends on the cluster mean of the level-one covariates $\bar{x}i,k(k=1,2,\ldots ,K)$, which picks up any correlation between this variable and the level-two error [55, 59, 60]. In both specifications $\beta_{k}^{w}(k=1,2,\ldots ,K)$ measures the within-cluster effect. Given that it is estimated using only within-cluster variation, in a linear specification, the estimate from both the within-between random model and correlated random-effects models are identical to the fixed-effects estimates [61–63]. $\beta_{k}^{b}$ measures the between effect and is estimated using only between-cluster variation whereas $\beta_{j}^{R}(j=1,2,\ldots ,J)$ measures the between effect for time-invariant variables.

The test of whether the between-cluster effect is significantly different from the within-cluster effect, *H*_0_: *β*^*b*^ = *β*^*w*^, is a Wald test. If the null hypothesis cannot be rejected, it indicates that the assumption of independence between the level-two error and the level-one covariates of the random-effects models holds, thus rendering a within-between random model to a standard random-effects model. In comparison with the Hausman test, the test still functions if the difference covariance matrices in the Hausman test is not positive definite, and works at the level of individual variables [60].

In comparison with the random- and fixed-effects models, a within-between random model differentiates within- and between-cluster effects. It provides fixed-effects estimates for level-one variables, allows inclusion of level-two cluster-invariant covariates and, in the linear case, it relaxes the assumption of zero correlation between the level-two error and the level-one variables so providing estimates that are robust to unobserved heterogeneity [54, 56, 58–61, 64].

Of interest here are the relationships between the exposure, alcohol consumption *AC*_*i*,*t*_, log household income, *lnY*_*i*,*t*_ and the outcome, affective theta *Θ*_*i*,*t*_. Specifically, the within-cluster effect, $\beta_{AC}^{w}$, assesses how on average a within-cluster change in alcohol consumption is associated with a within-cluster change in affect. In contrast, the between-cluster effect, $\beta_{AC}^{b}$, assesses how a change in cluster mean of alcohol consumption is associated with a change in cluster mean of affect. In this context, the compensating income variation (*CIV*) can be worked out in two components, a *CIV* for the within effect and one for the between effect, as follows:

$$CIV=Y*(\text{exp}\left(-\frac{\beta_{AC}}{\beta_{lnY}}\right)-1)$$ (8)

The within-between random model is applied within the umbrella of generalised linear mixed models [60, 65] and it is estimated using maximum likelihood. A test for the significance of *CIV* is a test of the statistical significance of exp(*-β*_*AC*_*/β*_*lnY*_)– 1, which is denoted by *τ*. Let **λ** be a (2, 1) vector of the estimates for alcohol consumption and household income ${\left[{\hat{\beta }}_{AC}\;{\hat{\beta }}_{lnY}\right]}^{\text{T}}$, Cov(**λ)** is the covariance matrix of **λ**, Cov(*τ*) the covariance matrix of *τ* and *g*(.) the transformation of **λ** into *τ*. Then the covariance of *τ* is obtained as follows:

$$Cov(\hat{\tau })=\left(\frac{\partial g(\hat{\lambda })}{\partial \lambda }\right)Cov(\hat{\lambda }){\left(\frac{\partial g(\hat{\lambda })}{\partial \lambda }\right)}^{T}$$ (9)

where

$$\frac{\partial g(\hat{\lambda })}{\partial \lambda }=\left[\begin{array}{cc} -\text{exp}\left(-\frac{{\hat{\beta }}_{AC}}{{\hat{\beta }}_{lnY}}\right)*\frac{1}{{\hat{\beta }}_{lnY}} & \text{exp}\left(-\frac{{\hat{\beta }}_{AC}}{{\hat{\beta }}_{lnY}}\right)*\frac{{\hat{\beta }}_{AC}}{{\hat{\beta }}_{lnY}^{2}} \end{array}\right]$$ (10)

The value of *CIV*, denoted by *CIV*^*£*^, is calculated based on the distribution of mean income before tax and mean tax by age range provided by the Office of National Statistics provide (taxpayers only; 2015 to 2016) [66]. For completeness, estimates from analogous, standard, random-effects, and fixed-effects models are also presented in Table 2.

**Table 2:** Regression equations for alcohol use, depression, neuroticism and household income.

	Cross-sectional		Longitudinal					
			Within-between random model		Fixed model		Random model	
	Depression (*t*)	Neuroticism (*t*)	Depression (*z*)	Neuroticism (*z*)	Depression (*z*)	Neuroticism (*z*)	Depression (*z*)	Neuroticism (*z*)
*Within Effect*								
Household Income			0.041 (2.15)	-0.005 (-0.32)	0.033 (1.61)	-0.005 (-0.32)		
Alcohol Consumption			0.000 (0.63)	0.001* (2.06)	0.001 (1.63)	0.001* (2.38)		
*Between Effect*								
Household Income	-0.157*** (-23.86)	-0.166*** (-32.02)	-0.146*** (-9.51)	-0.158*** (-7.44)			-0.076*** (-5.95)	-0.069*** (-5.57)
Alcohol Consumption	0.001*** (7.64)	0.001*** (9.24)	0.001* (2.32)	0.001* (2.33)			0.001 (1.92)	0.001** (2.93)
*Statistics*								
$\beta_{lnY}^{w}=\beta_{lnY}^{b}$ (P-value)				< 0.001				
$\beta_{AC}^{w}=\beta_{AC}^{b}$ (P-value)				0.9627				
Wald Chi2 (P-value)				< 0.001				
F (P-value)	< 0.001	< 0.001			< 0.001	0.153	< 0.001	< 0.001
R-squared	0.082	0.067						
Mean income (£)		30,528.11		30,486.57				
CIV (between effect)	0.671%	0.758%	0.584%	0.728%				
CIV (£)	204.89±0.06	231.26±0.04	178.04±1.52	222.03±0.87				

Note:

* p<0.05,

** p<0.01,

*** p<0.001

The value of *CIV* in sterling pounds, denoted by *CIV*^*£*^, is calculated based on the distribution of mean income before tax and mean tax by age range provided by the Office of National Statistics as follows:

$$CIV^{£}=CIV*\sum_{i=1}^{8}p_{i}*(income_{i}-tax_{i})$$ (11)

where *p*_*i*_ is the proportion of participants of the *i*^th^ age band out of the whole sample; *income*_*i*_ and *tax*_*i*_ are mean income before tax and mean tax reported by the Office of National Statistics in the *i*^th^ age band; *i* = 1, 2, …, 8 which correspond to age bands 35–39, 40–44, …, 70–74 years. Results are reported in Table 2.

## Results

### Sample characteristics

The means and standard deviations of depression *Θ*_*D*_ and neuroticism *Θ*_*N*_ and alcohol consumption by demographic are reported in Table 1. Further, Generalised Linear Models (GLM) were run on the whole sample to assess the associations between demographic characteristics and depression and neuroticism. The last two columns in Table 1 report the GLM results of demographic characteristics as a function of their *Θ*s. Both methods suggest similar sample characteristics. Referring to Table 1, socioeconomic status showed significant associations with affect as levels of both depression and neuroticism increases with deprivation. Those with a college education experienced less depression and neuroticism compared to participants with lower levels of education, as were those who were unemployed when compared to the employed. Respondents who consumed five or more portions of vegetables and fruits exhibited lowed levels of depression and neuroticism, as were non-smokers compared to smokers. Being obese or experiencing long-term illness was associated with a loss of affect. The number of children as one of the measures for family size showed a negative impact on *Θ*. Negative relationships between age and depression, and age and neuroticism were noted. Male respondents were typically less depressed or neurotic than female respondents.

### Cross-sectional analysis

Referring to Table 2, in the cross-sectional analyses greater income was significantly associated with lower measured depression and neuroticism. The cross-sectional average household income was £30,528 per annum. Each gram/day increase in alcohol consumption would require an increase in total annual household income of 0.67% (95% CI ±0.00019%), to offset the increase in depression *Θ*, indicating an increase of approximately £204.89 (95% CI ± £0.06) in annual household income. This effect replicates for neuroticism, each gram/day increase in alcohol consumption would require an increase in total annual household income of 0.76% (95% CI ±0.00014%), to offset the increase in neuroticism, indicating an increase of £231.26 (95% CI ± £0.04) in annual household income.

### Longitudinal analysis

Table 2 reports the results for depression and neuroticism. The coefficients for alcohol consumption and household income, $\beta_{AC}^{b}$ and $\beta_{lnY}^{b}$, give their between-cluster effects, which ostensibly address the question “what is the effect of a level-two individual moving from one level-two entity to another?” The significantly positive estimated coefficient ($\beta_{AC}^{b}$) indicates that a between-individual one-gram/day increase in alcohol consumption is associated with an increase in depression and neuroticism, suggesting that cohorts who drink more are more depressed or neurotic, $\beta_{lnY}^{b}$, whereas cohorts with a greater household income are less depressed or neurotic.

The longitudinal average household income is £30,486.57 per annum. In this context, the between *CIV*^*b*^, which is calculated using between-effect information, can be interpreted as compared to person one, who consumes X grams/day of alcohol, an increase in total household income of 0.58% (95% CI ±0.005%), approximately £178.04 (95% CI ± £1.52), is required to offset the increase in depression for person two who consumes X+1 grams/day.

In contrast, $\beta_{AC}^{w}$ and $\beta_{lnY}^{w}$ give the within-cluster effects, and they are concerned with what happens to outcomes when a level-one variable increases or decreases. Thus, the within *CIV*^*w*^ can be interpreted as when an individual consumes one gram/day more alcohol, how much more household income they will require to maintain the same level of depression. The estimated results indicate insignificant within-individual association between alcohol consumption and depression, and significantly positive association between household income and depression. However, given that in the longitudinal sample information of only two moments in time from the cohorts are applied the within-effect is not fully explored and do not provide meaningful information on our understanding of the within-effect. For neuroticism, the results replicate the longitudinal analyses. The between coefficients also show a significantly positive relationship between alcohol consumption and neuroticism, indicating that a between-individual one-gram/day increase in alcohol consumption is associated with an increase in neuroticism. Household income, on the other hand, has a significantly negative relationship with neuroticism. The results suggest a between *CIV*^*b*^ of 0.728% (95% CI ± 0.003%), which is equivalent to £222.03 (95% CI ± £0.87).

## Discussion

There were several notable findings from the present investigation. First, consistent with previous cross-sectional and longitudinal research, a robust relationship between alcohol consumption and affect was observed, and between income and affective state. Second, using the relationship between household income and negative affect, the shadow price for alcohol consumption per gram consumed was calculated, with broadly consistent results: for cross-sectional analyses, £204 (depression) and £231 (neuroticism), and for the within-between random model, £178.04 for depression and £222.03 for neuroticism. Averaging across these values yields a cost per gram of alcohol per day of £209.06 (95% CI £171.84 to £246.26) on annual household income. For those interested in the cost-effectiveness of alcohol policy, this estimate captures the intangible effects of alcohol and might be used to inform estimates of the societal costs of alcohol consumption [20].

The present study demonstrates the efficacy of applying shadow pricing methods to matters concerning alcohol-induced affective states. The results from cross-sectional and longitudinal modelling indicate that an increase in alcohol consumption of one gram each day has a significant effect on the consumers’ affective state. There is enough evidence to suggest policies that protect alcohol consumers’ presumed well-being by limiting initiatives that serve to reduce consumption are misaligned with the research evidence. Moreover, the analyses presented here are the first to estimate the value of an alcohol-induced deprecation to affect, required in order to optimise policy decisions to those that provide the greatest benefit [14].

This study has several strengths. First, the computed IRT theta metric is a reliable and validated measure. In comparison with a summated score, the theta is less likely to over-estimate repeated measure variance [45] and produces smaller sample bias [46]. Furthermore, IRT provides additional individual-scale, item-level information and recognises the multifaceted nature of affect. As a standardised score, the IRT theta provides a more stable and reliable construct over time in longitudinal analysis. Second, a within-between random model was conducted in the longitudinal analysis. In contrast to traditional random- and fixed-effects models, a within-between random model differentiates within- and between-cluster effects. It provides consistent estimates of within-cluster variation fixed-effects estimates but also allows inclusion of cluster-invariant covariates. More importantly, in the linear case, it relaxes the assumption of strictly exogenous regressors with respect to the idiosyncratic error component so providing estimates that are robust to unobserved heterogeneity [54, 56, 58–61, 64]. However, there were some limitations we should acknowledge. First, both fixed and random effects should not necessarily be interpreted as causal effects [55, 59, 60]. Although we are not looking for the direct, causal effect of alcohol consumption on negative affect, we see alcohol consumption as a proxy for a range of unmeasured drinking-related processes, such as drinking habit or drinking environment. It is feasible that unmeasured individual characteristics or externalities may attenuate or accentuate estimates. Second, while the weight of evidence suggests that directionality is from alcohol use to negative affect there is evidence that for some clinical groups alcohol is used to self-medicate [52]. As such, we conclude that, while these results are likely applicable to the majority of alcohol consumers, directionality may be reversed in some groups.

## Conclusion

The findings presented here are consistent with the research evidence suggesting that greater alcohol use is detrimental to consumers’ affective state and that policies that reduce alcohol consumption improve public well-being. Shadow pricing methods were used to assign value to the affective and intangible consequences of alcohol consumption. The affective benefit of reducing alcohol consumption by one bottle of wine (75g ethanol per 750ml) each week is equivalent to an increase in annual household income of £2,389 whereas reducing consumption by one pint of beer (20.45g ethanol per 568ml) each week is equivalent to an increase in annual household income of £610.69, and in addition to the purchase price of alcohol.
